# Low temperature and high field regimes of connected kagome artificial spin ice: the role of domain wall topology

**DOI:** 10.1038/srep30218

**Published:** 2016-07-22

**Authors:** Katharina Zeissler, Megha Chadha, Edmund Lovell, Lesley F. Cohen, Will R. Branford

**Affiliations:** 1 Blackett Laboratory, Imperial College, Prince Consort Road, SW7 2AZ, London, UK

## Abstract

Artificial spin ices are frustrated magnetic nanostructures where single domain nanobars act as macrosized spins. In connected kagome artificial spin ice arrays, reversal occurs along one-dimensional chains by propagation of ferromagnetic domain walls through Y-shaped vertices. Both the vertices and the walls are complex chiral objects with well-defined topological edge-charges. At room temperature, it is established that the topological edge-charges determine the exact switching reversal path taken. However, magnetic reversal at low temperatures has received much less attention and how these chiral objects interact at reduced temperature is unknown. In this study we use magnetic force microscopy to image the magnetic reversal process at low temperatures revealing the formation of quite remarkable high energy remanence states and a change in the dynamics of the reversal process. The implication is the breakdown of the artificial spin ice regime in these connected structures at low temperatures.

At room temperature and above, the magnetic reversal of artificial spin ice driven by magnetic field^1^,^2^,^3^,^4^,^5^ or thermal energy, is widely reported^6^,^7^,^8^,^9^,^10^,^11^,^12^,^13^,^14^,^15^,^16^,^17^,^18^. At zero field single domain bars can be treated as dumbbells, due to their Ising like structure (see Fig. 1(a,b)). Each bar is represented as a dumbbell of length *l* with oppositely charged magnetic charges, *q* = *M*_*s*_*tw*, at either end (where *M*_*s*_ is the saturation magnetization, *t* is the thickness and *w* is the width of the bar)^19^. Summing the three “dumbbell” charges meeting at each vertex results in the net vertex charge *Q* = *∑q* (see Fig. 1(b)). A vertex obeying the ice rule has a charge Q = ±1q whereas an ice rule violation vertex has a charge Q = ±3q (see Fig. 1d). At remanence and room temperature the majority of the artificial spin ice vertices will be in a Q = ±1q and rarely in a Q = ±3q state.

The occurrence of ice rule violations at room temperature is exceedingly rare (<1% in connected permalloy honeycomb array extracted from data published in)^5^. Their occurrence rate is related to the domain wall type. In permalloy systems two domain types are supported: transverse and vortex domain walls^20^. The magnetisation of a vortex domain wall rotates 360° about an out-off plane core whereas the magnetisation of a transverse domain wall rotates in plane resulting in the moments at the centre of the wall to point transverse to the saturated domains at either end. In permalloy systems such high energy states are only supported in transverse domain wall mediated reversal. Their rarity is ensured due to the creation mechanism which requires two domain walls to arrive at the same vertex without either wall propagating further into the array. This results in a total charge change of ΔQ = ±4q from the original saturated state (see Fig. 1(d))^1^; The ΔQ = ±4q ice rule violation is not observed in disconnected arrays^21^ or in arrays where switching occurs via vortex domain walls^4^,^22^,^23^. In comparison to transverse domain walls which have magnetic discontinuities at the bar edges and hence have sources of stray field, the 360° rotation of the magnetization of a vortex domain wall minimizes stray fields. This leads to vortex domain walls fitting the artificial spin ice model of moving magnetic charge more precisely which prohibits ice rule violations. In this model a magnetic charge arriving at a vertex creates a constructive field in addition to the external field which leads to the nucleation of a new domain wall and hence an reversal avalanche rendering ice rule violation unstable states^21^. Theoretical models describing dipolar needles on a kagome lattice suggest an increasing likelihood of violations with *increasing* temperature^24^,^25^. At room temperature the observation of ice rule violations is not impossible but are hardly observed in systems with low disorder. This leads to the conclusion that the permalloy artificial spin ice on the kagome lattice is in the Ice I phase as introduced by Möller *et al*.^23^ The Ice I phase is characterised by the individual bars being treated as dipolar needles approximated by a dumbbell. Each dumbbell can be be in one of two antiparallel states but no vertex can be in a state representing an ice rule violation.

Let us first review the reversal process at room temperature for transverse walls. Starting from a fully saturated spin ice state (see Fig. 1(a)) connected artificial spin ice reverses via the creation and movement of domain walls. The initial domain wall nucleation occurs at the array edges where the nucleation field is lower than that of the array bulk; domain walls traverse the array leaving one-dimensional chains of switched bars in their wake as shown in Fig. 1(c). The magnetic reversal, induced by an applied field parallel to one of the bar sub-lattices, is controlled by the ice rules^19^,^21^,^23^,^26^,^27^. Magnetic reversal which starts in a diagonal bar leads to the reversal of the adjacent vertical bar due to the external field pushing the domain wall through the connecting vertex. Reversing the vertical bar, aligned parallel to the external field, leads to an ice rule violation. The field required to force this high energy state is sufficient to reverse one of the subsequent bars and so the structure reverses in domino-style chain process once this field is reached. This leads to the existence of strong correlations between bars, and a characteristic reversal behaviour mediated by one dimensional chains^5^,^19^,^21^,^23^,^26^,^27^. If a direction of motion is imposed on the honeycomb lattice by applying a magnetic field, then there are two distinct vertex types, an ‘out-vertex’ where one wire branches into two and an ‘in-vertex’ where two wires merge into one. At every out vertex the domain wall must choose one of the two apparently identical paths at the Y-shaped junction. In fact the path chosen at the out-vertex is controlled by the domain wall chirality i.e. topology, independent of whether the system supports transverse domain walls^5^ or vortex domain walls^28^. The domain wall chirality refers to the handedness of the magnetisation rotation within the domain wall. However, the implied 100% path selectivity in the case of transverse domain walls is not experimentally observed due to the randomising effect of Walker breakdown which results in a change of the domain wall chirality and hence the path taken. Vortex domain walls are more resilient to randomising effects such that a 100% path selectivity can be achieved^28^,^29^.

The reversal process is quite different when the thermal energy of the system is reduced. We find that below 104 K the interaction between transverse domain wall and vertex creates an energy barrier, which prevents domain walls of a specific topology from entering the ‘in-vertex’ in the lattice, creating a previously unobserved new micromagnetic state. This additional pinning significantly increases the switching field and dominates the switching dynamics at and below 50 ± 1 K. In our experiments we deliberately aligned the magnetic field parallel to one of the sublattices. We find that the switching process deviates from one dimensional domino-like chains as the temperature is lowered. Instead at each out-vertex the switching of the initial, vertical, bar leads to a correlated switching of both diagonal bars resulting in a branching-like reversal. These observations indicate that there is a clear change in the outcome of the interaction of a domain wall with the micromagnetic landscape imposed by the geometry of artificial spin ice at each vertex at low temperature. This leads to a breakdown of the artificial spin ice as a viable model system for bulk spin ice.

The low temperature behaviour of artificial spin ice, is interesting. Theoretical prediction suggests that a long range ordered state should form due to long range interactions^17^,^24^,^25^. Recent experiments have highlighted that these underlying processes can be masked, in uncapped permalloy nanostructures, by an energy scale associated with exchange pinning which occurs when the native oxide layer becomes antiferromagnetic below 20 K^30^. Interactions between antiferromagnetic and ferromagnetic layers have shown to act as pinning sites for domain walls^31^.

In order to probe whether the array reverses via chains or via branching, images were taken at remanence throughout the magnetic reversal of the kagome artificial spin ice at 104 ± 1 K, 50 ± 1 K, 40 ± 1 K, 30 ± 1 K and 8 ± 1 K as shown in Fig. 2 (see Supplementary information for the whole reversal). Careful mapping of the changes in the magnetic contrast reveals the reversal processes of the array and their temperatures dependences.

To collate statistics on the reversal process as a function of temperature we define a chain to occur when a domain wall arrives at a vertex and subsequently exits with the reversal of only one other bar, leaving the third bar in its original state. In contrast we define branching to occur when a domain wall arrives at a vertex and then reverses the two connected, thus far not switched, bars in the same process. Note that the ‘snapshots’ imaged cannot distinguish a true branching process from an incremental switching; we can only observe that the remaining bars reverse in the same field step between images. Table 1 shows vertex counting statistics at a range of temperatures, showing the number of vertices at which chain reversal could be confidently assigned, and the number at which it could not. Chain reversal will naturally result in some vertices that cannot be assigned at the point where two chains meet. Therefore the presence of some vertices at 295 K and 104 K with no chain assigned is not indicative of branching behaviour. However chain reversal alone cannot reverse all the bars in an area resulting in a ferromagnetically aligned domain of the type observed at 50 K and below. At room temperature the chain reversal stage is followed by a second stage at higher fields where the remaining bars switch by nucleation of additional domain walls. The field interval between the chain and additional nucleation stages is typically 1 mT^4^, to 5 mT^4^,^22^. Thus the observation of large areas fully switching in a 0.1 mT step is strong evidence of a genuine branching process. Table 1 summarises the number of chains occurring and their length at various temperatures studied. Several observations can be made: It is striking that at 104 K, where chain behaviour is observed, the average chain length is dramatically shorter than at 295 K^5^. Out of a total of 26 chains observed at 104 K, 62% did not exceed a length of 1 (out-vertex) decision (see ref. 5 for methodology). As the temperature is decreased below 104 K the number of chains observed decreased drastically. Only 4 chains were observed throughout the reversal at 8 ± 1 K; An 85% reduction in comparison to the number of chains observed at 104 ± 1 K. The reversal at 8 K is no longer dominated by long reversal chains. The majority of the array reverses via branching. At low temperatures we see that the magnetic reversal occurs via the growth of two dimensional domains. Both diagonal bars reverse before the parallel bar (branching in) and reversal of the parallel bar causes subsequent reversal of both diagonal bars (branching out). It is interesting to note that Ice rule violations occur at 10% of the observed vertices at 8 ± 1 K.

In addition to this dramatic change in the field driven reversal behaviour of permalloy artificial spin ice, we frequently observe two new low-temperature remanence vertex states between 8 ± 1 K and 50 ± 1 K (Note that the magnetic contrast at 50 ± 1 K was imaged with a tip magnetized with opposite polarity with respect to the other temperatures; this is reflected in the observed contrast inversion of the vertices). Figure 3(a,b) show high resolution images of the two states and three of their permutations. The saturated state, highlighted in Fig. 3(a), as well as the initial vertex state resemble the low energy vertex state observed during the reversal at higher temperatures (simulated in Fig. 3(d–f)). The two cases can be distinguish by carefully analysing the reversal sequence taking into account the contrast changes of all the neighbouring vertices. In a magnetic reversal at room temperature it is possible, indeed typical, to only observe low energy ±q vertex states of the type shown is Fig. 3(e–g). In order to characterize these new low-temperature remanence states, we study the magnetic charge distribution using MFM line scans through these vertices at 8 ± 1 K (Fig. 3(c)). The calibration procedure to understand and interpret the magnitude of the MFM frequency shift utilises the line scan through an ‘ordinary’ ice rule vertex of charge +q, two-in-one-out (marked as ‘saturated’ in Fig. 3(a,c), purple line). Figure 3(a) shows three of the four possible permutations of one of the new low temperature vertex types. It is comprised of two discrete segments: an intense contrast slightly offset from the vertex was observed to occur next to a faint contrast of opposite polarity centred at the vertex. The line profile through the magnetic contrast of this state is plotted in Fig. 3(c) (orange line). The faintly contrasted segment situated at the vertex centre was found to have an identical MFM signal to the standard ice-rules vertex. The intense off-centre magnetic contrast has opposite sign and twice the magnitude of the MFM signal associated with an ‘ordinary’ ice rule vertex We interpret this as a ±2q magnetic object. The second observed vertex state is shown in Fig. 3(b). This vertex state shows two intense contrast segments of the same sign on either side of a faintly contrasted segment of opposite sign, which is situated at the centre of the vertex. Two permutations are possible (see Fig. 2(o)). The line profile through the state shown in Fig. 3(b) is shown in Fig. 3(c) (green line). As for the first vertex type, the faint contrast region at the centre has the same magnitude of MFM frequency shift as the ordinary ice rules vertex, and the off-centre intense contrast segments have twice the MFM frequency shift. The intense contrast can now be seen as a magnetic object of net charge ±2q and the faint contrast can be viewed as a magnetic object of charge 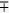 q (referred to as [±2q: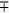 q] state) (schematics in see Fig. 3(a)). This leads to the identification of the state seen in Fig. 3(a) as having a net magnetic charge of Q = ±q and the state in Fig. 3(b) as having a net charge of Q = ±3q (schematics in see Fig. 3(b)). Hence overall we are looking at a |variation of ice rule states and ice rule violation states respectively. At 8 ± 1 K 38% of the vertices were observed to feature one of the new states. The new states are observed at the boundary between domains of switched vertices and not yet switched vertices. By solving the magnetic state in each sequential image we can determine the direction of domain growth. We observe that the [±2q: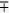 q] states occur at the in-vertices.

In order to understand the images more fully we perform micromagnetic simulations using OOMMF. The simulation reveals the ±2q magnetic objects to be transverse domain walls which are pinned as a consequence of their chirality. Figure 3(d) shows a down domain wall which was artificially injected into one of the diagonal bars and was then allowed to relax into a minimum energy state. The domain wall was absorbed into the vertex (Fig. 3(e)). The magnetic moment divergence (−∇. *M*) which is related to the vertex charge and the stray field emanating from the vertex is shown in Fig. 3(f) and is in good quantitative agreement to the MFM contrast (Fig. 3(g)). On the other hand, if an up transverse domain wall is injected (Fig. 3(h)) the minimum energy state observed differs. The domain wall was found to remain pinned just before the vertex leading to a contrast (Fig. 3(j)) as observed in the MFM images (Fig. 3(k)). In the micromagnetic simulations the two domain wall chiralities approaching from the diagonal bar are energetically equivalent at the starting point. The total magnetic energy in the system after relaxation, as calculated by OOMMF at 0 K, was found to be 2 × 10^−17^ J when no domain wall was pinned and 4 × 10^−17^ J in the case of a pinned domain wall confirming that the presence of a domain wall is a high energy metastable state. In wires parallel to the applied magnetic field the domain wall chirality during propagation is not conserved at fields above the Walker field. This is a process called Walker breakdown. This would reduce the domain wall pinning at the vertices greatly. However in the diagonal bars, Walker break down is suppressed due to the large field component parallel to the domain wall core^32^,^33^. In such a field arrangement the domain wall dynamics is rather complex; it has been observed previously that the domain wall preserves its chirality despite ‘shedding’ its antivortex core typically seen in ‘normal’ Walker breakdown^32^.

The observation of branching was found to be a result of the observed domain wall pinning in the diagonal bar. A field large enough to depin the domain wall from the in-vertex into the connecting vertical bar (above 40 mT at 8 ± 1 K) is large in comparison to the field necessary to push a wall through the adjacent out-vertex (Fig. 4(a–d)). The simulations shown in Fig. 4(e–h) show that at such high driving fields the behaviour at the ‘out’ vertex changes and both diagonal bars reverse (branching) via a complex sequence of vortex like domain walls (see movie in Supplementary material).

The greater stability of the [±2q:
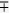
q] vertex states play a leading role in understanding both the transformation from a reversal via one dimensional chains to two dimensional branching and the increased population of ice rule defects. The branching is a consequence of the increase in the reversal field as the pinning increases. There is always a competition between the Zeeman energy which favours reversal of all bars and the energy barrier to the nucleation of an additional domain wall, which gives the chain reversal. The mechanism for ice rule defect formation at room temperature in transverse wall regime relies on the mutual Coulombic repulsion of two domain walls simultaneously approaching the same in-vertex^1^. This is a rare event because the wall will transit the full length of the nanobar in a single field step. Below 50 K we see the domain wall pinned at the in-vertex over a number of field steps and so there is much more opportunity for a second wall to arrive and set up a double domain-wall state.

The domain wall chirality in the diagonal bars is set by the external field which aligns the domain wall magnetization parallel to the applied field and annihilates any unfavourable domain walls at the edges. The thus favoured domain wall is pinned at the adjacent in-vertex. The low energy vertex state cannot be reached due to the incompatibility between the domain wall and vertex topology. In other words, the magnetic moment orientation at the vertex, set by the initial saturation and the geometry, is 180° rotated with respect to the domain wall core moments. The high energy state is stabilised via a competition between the magnetostatic attraction of the domain wall charge and the vertex charge, which are of opposite sign, and the repulsion caused by the exchange interaction which favours the gradual unwinding of the magnetisation twist in order to achieve a parallel moment arrangement. This competition of attractive and repulsive interactions effectively creates an energy barrier for the domain wall. Above the crossover temperature which separates the one dimensional avalanche regime and two dimensional branching regime instead of stabilizing the metastable high energy vertex state thermal energy allows a relaxation into the lower energy state via the rapid rotation of the domain wall core magnetic moments. Energy has to be applied to the system in order to push the domain wall over the imposed energy barrier and into the low energy vertex state. There are mechanisms which transfer energy to the magnetic moments: external magnetic fields and temperature.

Previous experimental studies into the temperature activated transformation of one domain wall type into another have found an energy barrier of the order of 10^−21^ J^34^. As the temperature is lowered the critical external magnetic field strength, needed to push the domain wall over the barrier, increases. In our experiments, we find that between 50 ± 1 K and 104 ± 1 K the applied field strength, needed to facilitate the reversal, triggers a drastic change in the reversal dynamics. At higher temperatures thermal energy is enough to spontaneously relax the high energy state into the low energy state. Similar temperature dependent behaviour of domain walls has been observed before. At high temperatures (533 K and 583 K) in wires whose dimensions are close to the phase boundary between transverse domain walls and vortex domain walls spontaneous switching between the domain wall types was observed without a biasing field^34^. The application of an external magnetic field can lower critical temperature scales in magnetic materials^35^,^36^. The stochastic depinning of a vortex domain wall from a single notch in a permalloy wire is sensitive to the temperature as well as biasing field strength and was found to occur via metastable states above 30 K^36^. Below 30 K a two state model (pinned versus unpinned) separated by a single energy barrier could be observed^36^. The origin of the drastic change in the chain occurrences, 17% to 4%, below 30 K is still unclear. Wuth *et al*.^36^, show that at low temperatures domain walls depin via a sharp transition, i.e. the domain wall overcomes a single energy barrier. In the context of artificial spin ice this translates to an ice rule violation being a state where two domain walls are pinned in front of the vertex (one in each bar) and each wall is prevented from proceeding into and through the vertex by an energy barrier. Wuth *et al*. are less clear as to the origin of the complex energy landscape, however they state above 30 K this becomes more significant. They observe time dependent switching above 30 K which they attribute either to the existence of multiple energy barriers or to the existence of numerous metastable states during the overcoming of a single energy barrier. Indeed perhaps these two pictures are in some sense the same.However, it is important to note that in uncapped permalloy wires exchange bias effects due to the oxide layer becoming antiferromagnetic below 20 K can potentially influence the pinning and depinning of ferromagnetic domain walls^30^. It has recently been shown that domain walls in a ferromagnetic wire can be pinned using a superimposed antiferromagnetic layer^31^. However the persistence of the observed features up to 50 ± 1 K suggests an origin which extends beyond the pinning introduced by the native oxide. In this case we propose that the domain wall topology is the likely cause of the enhanced pinning in our experiments.

At low temperatures and at high magnetic fields the artificial spin ice regime breaks down. However the control of domain wall motion in nanoscale systems has wide implications for commercialization such as race track memory applications^37^,^38^ as well as for example, domain wall driven movement of surface-functionalized superparamagnetic microbeads for biological and chemical use^39^,^40^. It may be desirable to change the reversal characteristics of magnetic junctions in a controllable fashion in domain wall based logic devices. The processes our experiments have uncovered could be realised at higher temperatures if pinning strength were increased by introduction of artificial pinning layer of the sort used routinely in spin valve architectures that would produce pinning at elevated temperatures, facilitating the existence of these new exotic ice rule defects for room temperature operation. The out-vertices act as a signal router in the high temperature regime^28^ and a signal duplicator in the low temperature regime^41^. Interestingly if the effect could be turned on and off, for example by a magneto-electric effect with local gating^42^,^43^, then the effect at the out-vertex could be utilised to provide reprogrammable domain wall logic functions.

## Method

### Fabrication

The honeycomb artificial spin ice was fabricated from permalloy (Ni80Fe20) using conventional electron-beam lithography, followed by thermal evaporation of the metal from a Ni81Fe19 target and lift-off. The lithographic template was fabricated using a single 300 nm thick layer of 950 A4 polymethyl methacrylate. The developed pattern was plasma ashed in oxygen plasma using an Emitech K1050X Plasma Asher at 10 W for 2 min to remove residual resist. The artificial spin ice was fabricated on (100) silicon with an electrically insulating 300 nm oxide layer at the surface. The individual connected bars making up the honeycomb artificial spin ice were 120 + 5 nm wide, 1072 + 10 nm long and 18 + 2 nm thick.

### Magnetic force microscopy

The low temperature magnetic force microscopy images were obtained with an Atomic Force Microscope attoAFM/MFM Ixs in combination with an Oxford Instrument cryostat. The microscope was situated between the pole pieces of a split coil 4.0 T superconducting magnet. The magnetic force microscopy (MFM) was carried out at remanence after the application of successive in-plane magnetic fields. The field was ramped at a rate of 0.1 T/s while the tip was disengaged from the sample in steps of 1 Oe (field increment was 2 Oe in the case of 8 K data). The array was initially fully magnetized along the x direction and then small incremental fields of opposite polarity were applied. This allowed the mid reversal imaging of the array. The 10 μm × 10 μm images were acquired in a central area of the array. The MFM images were taken using the phase locked loop mode which tracks the resonance frequency (f_0_) shift of the cantilever which is directly proportional to d^2^B_z_/dz^2^ where B_z_ is the magnetic stray field of the sample which in turn is related to the total magnetic charge at the vertex (Q). An AppNano MAGT-HM high moment tip, with a coercivity of 50 mT, was used in tapping mode at a constant lift height of 120 nm. No atomic force microscopy AFM was performed in between the MFM images. The position, i.e. the drift, was monitored by non-magnetic dirt visible in the MFM due to a variation of height in comparison to the array.

### Micromagnetic simulations

**Figure 4:**
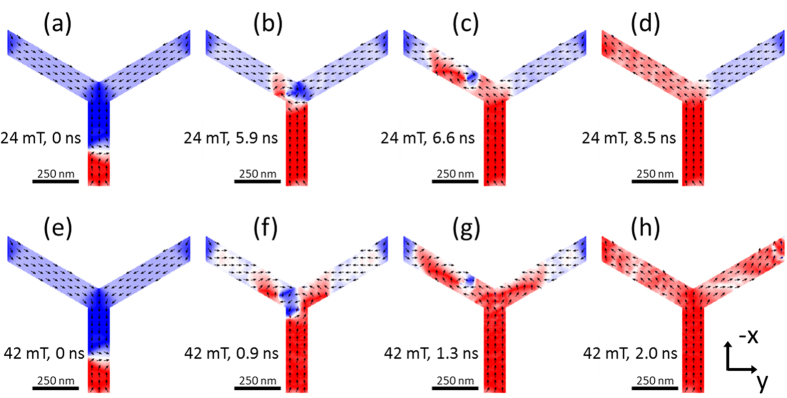
(**a**–**d**) and (**e–h**) Show snapshots of the simulated dynamic response of a down transverse domain wall in a 120 nm wide wire (initial state at 0 ns) under the application of a 24 mT or a 42 mT field applied along −x direction. Simulations reveal the origin of the branching mechanism. A transverse domain wall from the vertical bar causes the reversal of both diagonal bars providing a large enough driving force is applied. The experimentally observed pinning of domain walls in the diagonal bars results in domain walls experiencing a much larger field when they traverse the vertical bar and hence their dynamics changes leading to branching.

Micromagnetic simulations, undertaken to help the interpretation of the experimental results, were done using the Object Oriented Micromagnetic Framework (OOMMF)^44^. A Single vertex with bar width of 120 nm was simulated. The simulation framework assumes a temperature of 0 K. The saturation magnetisation and exchange constant of permalloy was taken to be 0.8 MAm^−1^ and 13 × 10^−12^ Jm^−1^, respectively. The minimum energy configuration of the relaxed state at zero field was calculated using a mesh size of x = 2 nm, y = 2 nm and z = 9 nm and a Gilbert damping parameter of α = 0.5 was used in all simulations. The domain walls were artificially injected using a colour map via a script vector field. The dynamic behaviour of a domain wall with respect to the driving field strength (applied along −x direction, see Fig. 4) was simulated using an x = 4 nm, y = 4 nm and z = 18 nm mesh and a damping parameter of α = 0.01.

### Data Availability

Data requests should be addressed to dataenquiryexss@imperial.ac.uk.

## Additional Information

**How to cite this article**: Zeissler, K. *et al*. Low temperature and high field regimes of connected kagome artificial spin ice: the role of domain wall topology. *Sci. Rep.* **6**, 30218; doi: 10.1038/srep30218 (2016).

## Supplementary Material

Supplementary Information

Supplementary Video 1

Supplementary Video 2

Supplementary Video 3

Supplementary Video 4

## Figures and Tables

**Figure 1:**
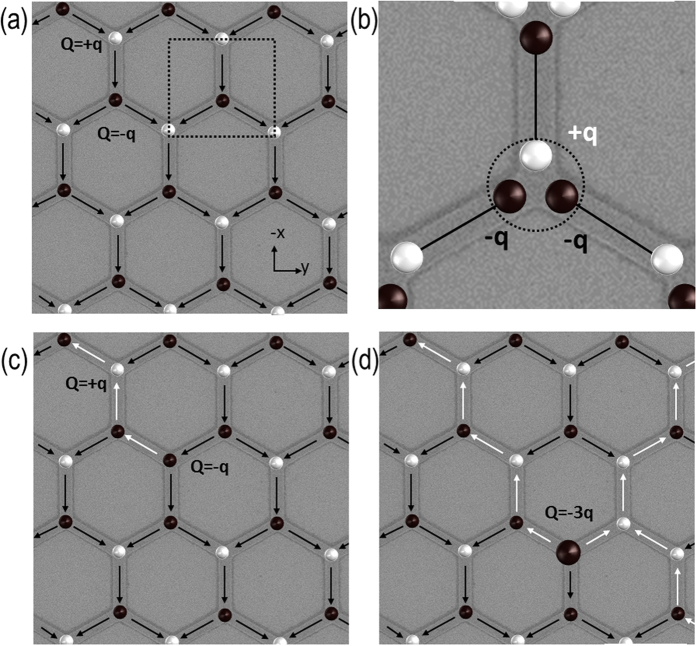
Schematics of the magnetic contrast in artificial spin ice. (**a**) Shows a saturated artificial spin ice. (**b**) A breakdown of the charge of the vertex highlighted in (**a**) using the dumbbell model. The vertex net charge, Q, is derived from the sum of the charges, ±q, at the end of each bar. Each vertex has a net charge of Q = ±q which translates to a dark or bright contrast area at the vertex in Magnetic Force Microscopy images. (**c**) The charge distribution of an artificial spin ice after a chain of bars has reversed. (**d**) Ice rule violation formation as previously observed in permalloy artificial spin ice. The two reversal chains terminate at the same vertex leading to a pinning of the two domain walls just before the vertex. At room temperature the formation of this excited state is exceptionally rare (around 1%).

**Figure 2:**
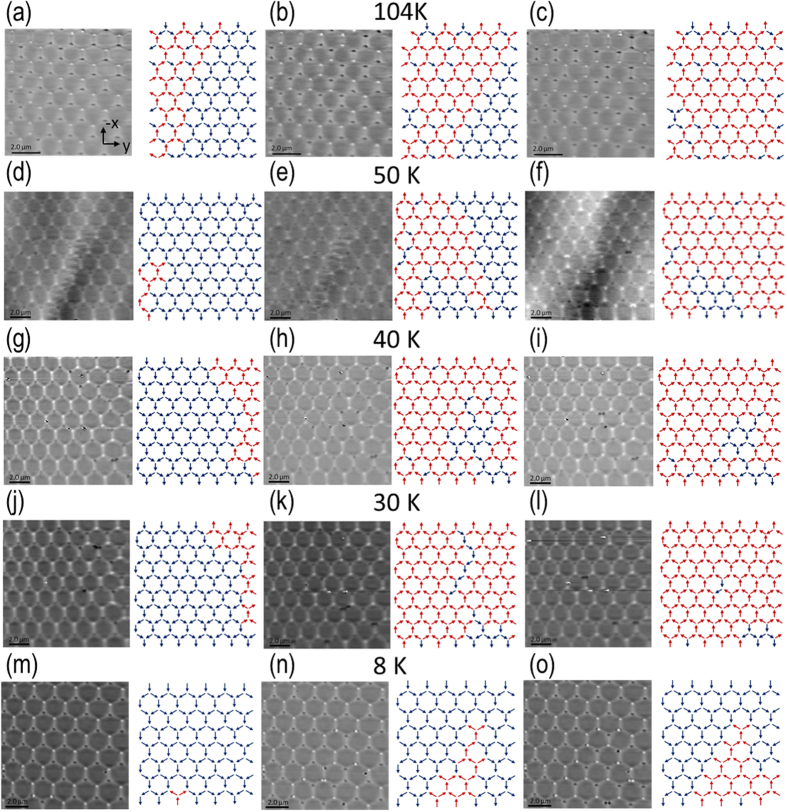
Magnetic force microscopy images throughout the magnetic reversal of the permalloy honeycomb artificial spin ice. The images were taken at remanence after the application of an incremental applied field along −x (blue arrows indicate initial state). Images were taken at 104 ± 1 K (**a**) −24.8 mT, (**b**) −25.6 mT, (**c**) and −25.8 mT, at 50 ± 1 K (**d**) −28.4 mT, (**e**) −28.7 mT and (**f**) −28.9 mT, at 40 ± 1 K (**g**) −29.9, (**h**) −30.0 and (**i**) −30.1 mT, at 30 ± 1 K (**j**) −31.0 mT, (**k**) −31.1 mT and (**l**) −31.2 mT and at 8 ± 1 K (**m**) −41.6 mT, (**n**) −42.2 mT and (**o**) −42.6 mT. The array reverses via domain wall movement (red arrows indicates a reversed bar). Unusual magnetic contrast around the domain boundaries was observed at 50 K and below. No such contrast was observed at 104 K.

**Figure 3:**
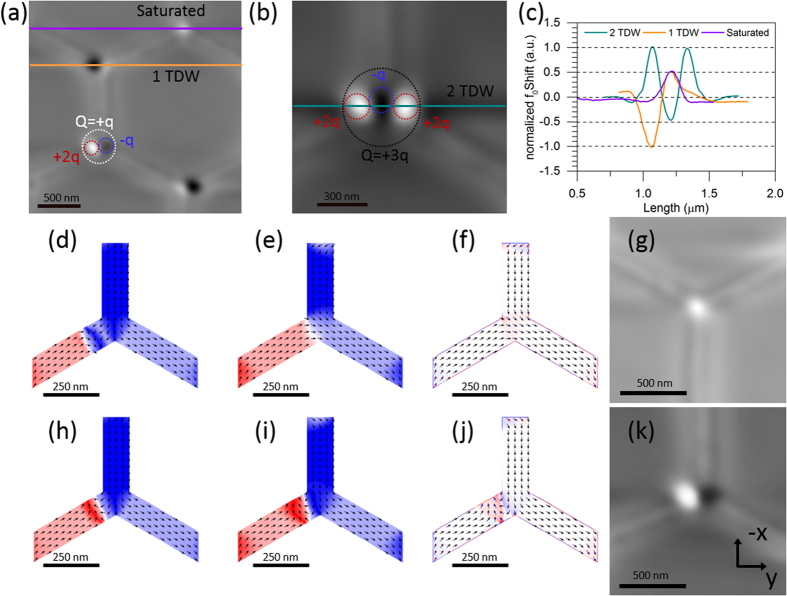
(**a**,**b**) Show high resolution magnetic force images of the two low temperature vertex states. (**a**) Shows 3 permutation of the first newly identified feature. It is characterized by a faint magnetic force signal (resonant frequency shift) at the vertex centre in conjunction with opposite polarity contrast of twice the magnitude just offset from the vertex. The enclosed area shows the composition of the Q = +q new low temperature remanence state [+2q:
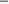
q]. (**b**) Shows one permutation of the second feature occurring at low temperature which features a faint magnetic force signal at the vertex centre surrounded by two high contrast features of, twice the magnitude and of opposite polarity to either side of the vertex. The enclosed area shows the composition of the Q = +3q new low temperature remanence state [+2q:
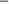
q:+2q]. (**c**) Shows the normalized line profiles through the features as shown in (**a**,**b**) as well as a line scan though a saturated vertex. The zero field micromagnetic state of a single vertex (**e**) was simulated using OOMMF after artificially injecting a down transverse domain wall. The initial, pre relaxation state is shown in (**d**,**f**) Shows the divergence of the magnetization in (**e**) which is of quantitative agreement with the MFM contrast imaged shown in (**g–k**) Show the same process as in (**d–g**) after the injection of an up transverse domain wall. The latter resulted in the pinning of the transverse domain wall just before the vertex.

**Table 1:** Comparison between the chain lengths observed and the change in the number of vertices involved in reversal chains at different temperatures.

Temperature (K)	Effective H_C_ (mT)	Vertex observations				Chain length (Number of Decisions)				
		Total vertices	Vertices not in chains	Vertices in chains	Ice rule violations	1	2	3	>3	Max chain length observed
295 ± 1^5^	10.0	768	282 (37%)	486 (63%)	7	5	34	28	27	9
104 ± 1	25.6	91	43 (47%)	48 (53%)	0	16	9	1	0	3
50 ± 1	28.7	104	91 (88%)	13 (12%)	4	13	0	0	0	1
40 ± 1	30.0	104	86 (83%)	18 (17%)	4	18	0	0	0	1
30 ± 1	31.1	104	100 (96%)	4 (4%)	7	4	0	0	0	1
8 ± 1	42.6	92	88 (96%)	4 (4%)	9	4	0	0	0	1

The room temperature numbers were extracted from the data published in ref. 5. The same methodology as published in ref. 5 was used in order to define the chains. ‘Vertices not in chains’ refers to vertices where a chain could not clearly be assigned. A clear trend to shorter chains was found as the temperature decreases. At 8 ± 1 K almost no chains are formed. Effective coercive field (H_C_) is defined as the first field at which at least half the bars have reversed.
