# Supplementary material for: Low temperature and high field regimes of connected kagome artificial spin ice: the role of domain wall topology
Source: Sci Rep. 2016 Jul 22;6:30218. doi: 10.1038/srep30218 (PMC4957146; doi:10.1038/srep30218)
Supplement: Supplementary Information [file srep30218-s1.doc]

Supplementary information for paper “Low temperature and high field regimes of connected kagome artificial spin ice: the role of domain wall topology.”

Katharina Zeissler^1^, Megha Chadha^1^, Edmund Lovell^1^, Lesley F Cohen^1^ and Will R Branford^1^

^1^*Blackett Laboratory, Imperial College, Prince Consort Road, SW7 2AZ, London, UK*

Video Legends for Supplementary Videos

Supplementary Video 1: Video of MFM images at selected fields during field driven magnetic reversal at 8K.

Supplementary Video 2: Video of MFM images at selected fields during field driven magnetic reversal at 30K.

Supplementary Video 3: Video of MFM images at selected fields during field driven magnetic reversal at 40K.

Supplementary Video 4: Video of MFM images at selected fields during field driven magnetic reversal at 50K.
